# Ultrasound and Infrared-Based Imaging Modalities for Diagnosis and Management of Cutaneous Diseases

**DOI:** 10.3389/fmed.2018.00115

**Published:** 2018-04-25

**Authors:** Sheliza Halani, F. Stuart Foster, Maksym Breslavets, Neil H. Shear

**Affiliations:** ^1^Faculty of Medicine, University of Toronto, Toronto, ON, Canada; ^2^Medical Biophysics, Sunnybrook Health Sciences Centre, Toronto, ON, Canada; ^3^Dermatology, University of Toronto, Toronto, ON, Canada

**Keywords:** non-invasive imaging, ultrasound, optical coherence tomography, reflectance confocal microscopy, dermatology, hidradenitis suppurativa, electrical impedance spectroscopy, spectrophotometric intracutaneous analysis

## Abstract

Non-invasive bedside imaging tools are becoming more prevalent for assessing cutaneous lesions. Ultrasound used at specific frequencies allows us to assess margins of lesions to minimize the extent of the biopsy that is performed and improve cosmetic outcomes. Vascularity, seen on Doppler ultrasound and contrast-enhanced ultrasound, and stiffness, assessed on tissue elastography, can help differentiate between benign and malignant lesions for clinicians to be more judicious in deciding whether to biopsy. Moreover, research has shown the efficacy in using ultrasound in monitoring flares of hidradenitis suppurativa, a disease affecting apocrine gland-rich areas of the body, for which the current gold standard involves examining and scoring inflammatory lesions with the naked eye. Infrared-based modalities have also been on the uptrend to aid in clinical decision-making regarding suspiciousness of lesions. Reflectance confocal microscopy has lateral resolution that is comparable to histopathology and it has been shown to be an appropriate adjunctive tool to dermoscopy, specifically when evaluating melanomas. Optical coherence tomography has utility in determining lesion thickness because of its depth penetration, and spectrophotometric intracutaneous analysis is becoming more popular as a tool that can be used by general practitioners to know when to refer to dermatology regarding worrisome pigmented lesions. Strides have been made to incorporate electrical impedance spectroscopy alongside dermoscopy in decision-making regarding excision, although the evidence for its use in the clincial setting remains inconclusive. This paper reviews the efficacy and drawbacks of these techniques in the field of dermatology and suggests future directions.

## Introduction

In the assessment of a patient with a cutaneous lesion or disease, one of the biggest quandaries is access to a bedside tool to assess extent of disease, malignancy of the lesion, and to plan management. One of the main goals of non-invasive imaging is to establish a diagnosis with high sensitivity and specificity compared with histopathology which is the current gold standard. Non-invasive bedside tools also allow for assessment of tumor thickness and borders to aid in decision-making for surgical excision or non-invasive therapy. Finally, they are valuable in monitoring treatment sites non-invasively so as to avoid repeated biopsies and scarring (1). This review discusses recent research in ultrasound, infrared (IR)-based modalities such as reflectance confocal microscopy (RCM), optical coherence tomography (OCT), and spectrophotometric intracutaneous analysis (SIAscopy), and electrical impedance spectroscopy (EIS) for the diagnosis and management of dermatologic diseases.

## Science and Vocabulary

All of the modalities discussed here are non-invasive and allow for real-time visualization of cutaneous structures. Table 1 [adapted from Ref. (2)] provides an overview of each of their characteristics.

**Table 1:** Comparison of imaging parameters for non-invasive techniques in dermatology.

	High-definition optical coherence tomography (OCT)	Conventional OCT	Reflectance confocal microscopy	High-frequency ultrasound	Spectrophotometric intracutaneous analysis	Electrical impedance spectroscopy
Input	Near-infrared (IR) light	Near-IR light	Near-IR light	Sound waves	Visible and IR light	Alternating current
Wavelength	1,300 nm	930–1,300 nm	445, 658, 785 nm	18.8–125 µm	400–1,000 nm (3)	1 kHz to 2.5 MHz (4)
Lateral resolution (μm)	3	7.5–15	1.25	60–250	(Similar to dermoscopy)	N/A
Axial resolution (μm)	3	5–10	3–5	30–120	(Similar to dermoscopy)	N/A
Penetration depth (mm)	0.5–1.0	1.5–2.0	0.25–0.30	4–30	2.0 (5)	(Four different depths/colors) (6)
Field of view (mm)	1.8 × 1.5	6.0 × 6.0	0.50 × 0.50	12.0	24.0 × 24.0 (12.0 × 12.0) (7)	5.0 × 5.0 (8)
Approximate time for imaging each site (min)	0.5	0.5	2 (for Vivascope^®^ 3000) 10 (for Vivascope^®^ 1500)	0.5	Seconds (9)	<0.17 (6)

*Modified table from Ref. (2)*.

### Ultrasound

Ultrasound is a modality that uses sound waves emitted by a transducer probe which is in contact with the skin. As they reflect off structures with different depths, the sound waves return to the probe with varying intensities and they are recorded by a processing unit and displayed on a screen (10). There are several modes, the most common one being B-mode in which 2D images are created in either longitudinal or transverse orientation (10). The frequencies of the ultrasound waves are measured in Mega-hertz, are inversely proportional to the wavelength, and range between 7.5 and 200 MHz for visualization of epidermis, dermis, and subcutaneous structures; the higher the frequency the higher the image resolution but the penetration depth decreases (10, 11). The ultrasound transducer, which is the handheld device that converts the electrical signal to ultrasound and vice versa, contains a range of frequencies, termed bandwidth (12). A broader bandwidth means that the transducer contains more than one frequency at which it operates, and the axial resolution is determined by the bandwidth of the pulse (13). The center frequency is the frequency at which the transducer is most sensitive (13).

### IR-Based Modalities

Reflectance confocal microscopy is a technique that uses near-IR light from a point laser light source (14). As this light passes through various cellular structures with differing refraction indices, the light is reflected from various skin structures and those with more melanin have more reflection (14). The backscattered photons create a two-dimensional image—the contrast that results is due to the refraction indices and sizes of various organelles and microstructures (15). This offers cellular resolution and horizontal sectioning (14–16). RCM has favorable lateral resolution, allowing visualization of structures on the cellular level. The field of view of an image is 0.5 by 0.5 mm^2^ and can be increased to 8 by 8 mm^2^ when a set of images is acquired and combined (15). This allows for tissue sampling of a wider region and the ability to pick up malignancy in a tumor that is sparse. This increases the sensitivity in comparison to a biopsy sample of a single site (15).

Optical coherence tomography is another non-invasive approach to producing real-time cross-sectional images of the skin using near-IR light (2). OCT is most useful for determining lesion thickness as this its depth of penetration is up to 2 mm compared with RCM which is approximately 0.25–0.3 mm (2). A specific form of OCT called high-definition optical coherence tomography (HD-OCT) uses IR light in the wavelength of 1,000–1,700 nm (2). HD-OCT has a lateral resolution of 3 µm compared with the 7.5–15 µm for conventional OCT (17).

Spectrophotometric intracutaneous analysis is another technique for imaging pigmented skin lesions that relies only on visible and near-IR light (18). When light hits the skin, each component reflects and absorbs light differently (7). A reflectance spectrum is created which depends on the histological composition of the skin and its chromophores such as melanin, blood, and collagen (18). The images that results indicate the distribution of these components (18).

### Electrical Impedance Spectroscopy

The final technique that is discussed in this paper is EIS. This technique determines the impedance of the scanned tissue by applying an alternating electrical current (19). It creates a score regarding the regularity or irregularity of cells and can help to differentiate between benign tissues which are more orderly, and malignant tissues which have more disorder (19, 20).

## Ultrasonography

The first sub-section proposes a multi-modal ultrasound approach to delineate between benign and malignant cutaneous lesions. Section “Can Ultrasound be Used for Diagnosis of and Evaluating Extent of HS?” consists of a special focus on hidradenitis suppurativa (HS) as research has introduced ultrasound as an imaging technique which can be used to assess extent of the disease rather than examination with just the naked eye.

### Ultrasonography of Suspicious Lesions: Benign Versus Malignant

Studies in the recent decade have elucidated the use of ultrasound as an adjunct in planning before surgical excision of skin lesions—localization of anatomical variations is key for invasive procedures to avoid the so-called *danger zones* (21). With the introduction of various ultrasound techniques, several parameters and characteristics of lesions can be depicted before planning for excision.

#### Depth of Lesions

Several studies have examined the use of ultrasound for identifying accurate margins before the surgical removal of cutaneous melanomas and for assessing the extent of a tumor and its degree of invasiveness. Maj et al. (22) showed that high-frequency ultrasound (HFUS) clearly identified melanomas (hypoechoic lesions) that were thicker than 1 mm, and they found that a statistically significant correlation existed between the Breslow score on histology and the depth of the tumor assessed on ultrasound preoperatively. This is important because the Breslow thickness is the most important prognostic factor in melanoma patients (22). Other authors (10) found with a 20-MHz ultrasound that HFUS was more fruitful than incisional biopsy to assess infiltrative parts of the tumor especially in mixed-component tumors. A retrospective analysis of melanomas found that with a 15-MHz probe, cutaneous ultrasound would properly stage the tumor and allow a single surgical intervention to be planned in approximately 80% of melanomas (23).

One of the pitfalls that have been reported by this group includes the overestimation of the depth of melanomas because of lymphocytic infiltration or naevus remnant (23). Literature shows that there is a higher sensitivity for thicker melanomas (depth greater than 1 mm), whereas the depth assessed on ultrasound in thinner melanomas may not have as strong of a correlation with histological depth (24). The higher frequency probes, such as 75 MHz, have been reported to more accurately classify Breslow thickness (25). For the 45 melanomas classified in a study by Guitera et al., none of the melanomas with a Breslow depth less than 1 mm were classified as greater 1 mm. The *in situ* melanomas were overestimated in 11 cases but still correctly classified as less than 1 mm (25).

While the higher frequencies allow for a greater accuracy of Breslow measurement in melanomas, this compromises penetration depth. Kučinskienė et al. (26) found that in basal cell carcinomas (BCCs), the range of frequencies between 13 and 15 MHz would allow for better visualization in tumor borders in BCCs. In BCCs with a thickness less than 1 mm, the Spearmen correlation coefficient between ultrasound and tumor depth was 0.860 in this study (26).

**Figure 1:**
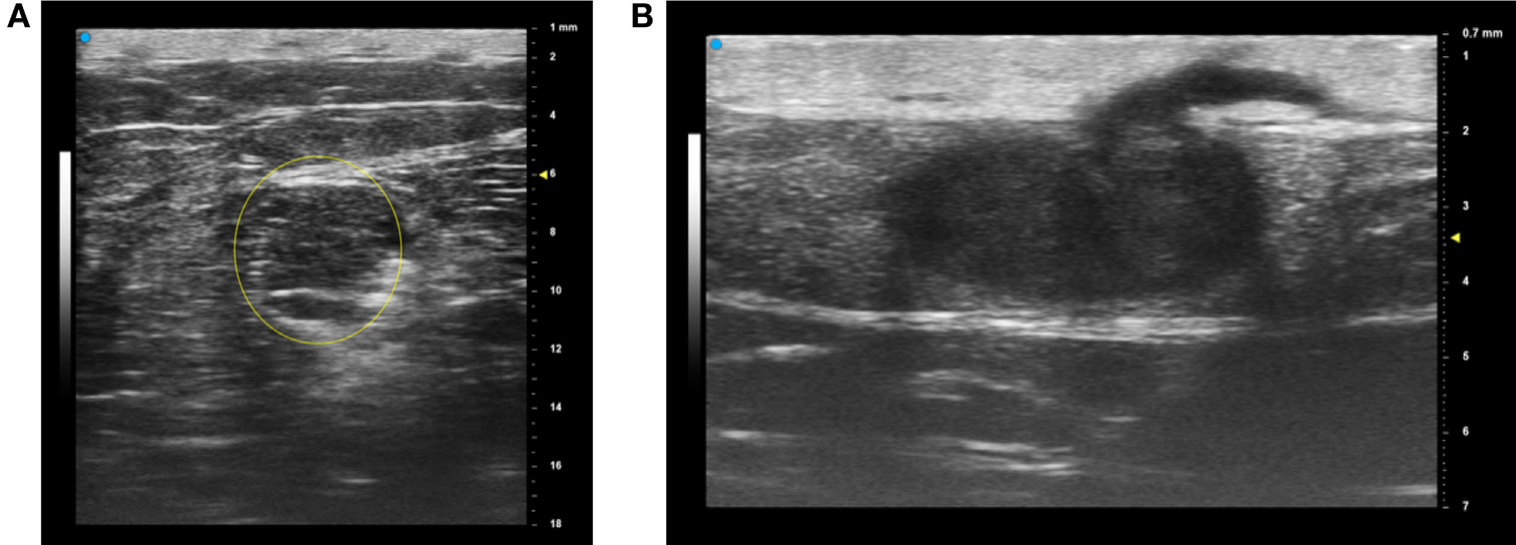
The most recent array-based ultrasound technologies for dermatology provide a choice of operating frequency, which enables the operator to “trade off” the field of view and resolution. For example, a deeper lesion (lipoma) is best imaged with a lower center frequency of 30 MHz **(A)** while a more superficial lesion and hair are better imaged at a center frequency of 50 MHz **(B)** with higher resolution but a smaller overall field of view. These images are courtesy of VisualSonics. Permissions obtained to reprint from Vevo MD for Dermatology (27), retrieved from https://www.visualsonics.com/product/imaging-systems/vevo-md Copyright 2017 Fujifilm VisualSonics Inc.

The newest array-based ultrasound technology from Fujifilm VisualSonics Inc. (VevoMD) offers the ability to select a frequency of operation from about 15 to 55 MHz center frequency (bandwidth from 12 to 70 MHz). Therefore, this is suitable for examining both superficial and deeper structures. For deep lesions such as the lipoma shown in Figure 1A, the selection of a lower center frequency (30 MHz) results in greater penetration and a larger field of view (15 mm by 18 mm in this case). A more superficial lesion and hair follicle can be imaged at much higher resolution by operating at 50 MHz as shown in Figure 1B, but the field of view is only 10 mm laterally by 7 mm deep. Increased image resolution is obtained with a higher operating frequency at the expense of the field of view (28). Using ultrasound, different frequencies can assist with assessing margins of various cutaneous lesions; however, more information is still needed for diagnostic accuracy.

#### How Do We Estimate the Vascularity of Lesions?

The vascularization of lesions is analyzed *via* color Doppler ultrasound and used in conjunction with the depth to assess a lesion. Dybiec et al. (29) described two case reports in which a benign cavernous hemangioma and melanoma were evaluated with ultrasound. The former was well-delimited, hypoechoic, and well-vascularized on HFUS while the latter was poorly delimited, irregular, hypoechoic, crossing the dermoepidermal junction and vascular signals appeared 6 months later (29).

Another way to examine vascularity is with contrast-enhanced ultrasound (CEUS). This technique involves a bolus of microbubbles that is given to a patient *via* an intravenous line and filming the lesion with the ultrasound thereafter. Both qualitative and quantitative data are taken to characterize the lesion as the injection passes through the lesion (30). Examples of parameters that are measured include the time for the tumor to uptake the contrast, the time from injection to maximal uptake (or peak), the time for wash out (from peak to eliminating the contrast), and the total time the contrast is visible (from injection to elimination) (31).

Stramare et al. (30) analyzed 14 patients using a 5.0–7.5 MHz probe and CEUS in 14 patients. All seven of the malignant lesions and all seven of the benign lesions were appropriately classified as there was a significant difference between the time to peak of the contrast between the two groups of lesions. Petik and Yildiz (32) found vascular signals in 13 of 14 malignant skin tumors (MST) and in 20 of 66 benign skin tumors (BST) with color and power Doppler. The BST had either mixed or peripheral circulatory types (but none with only central blood flow). The MST, on the other hand, consisted of either the peripheral, central, or mixed blood flow types. The peak systolic velocity and end diastolic velocity were higher in the MST group, especially for squamous cell carcinomas (SCCs) (32).

Detection of vessels in a skin lesion generally increases the suspicion for malignancy. Despite this, Scotto di Santolo et al. (33) highlighted in a retrospective analysis using color and power Doppler that while the vascular signal shows appropriate sensitivity and specificity in identification of malignant lesions, the pitfall of these modalities is that these cannot be used as an absolute marker since other lesions display high vascularity. Power Doppler is a technique in which the strength of the Doppler signal is displayed rather than the speed and direction of the signal (34). In one study, 25% of benign lesions such as inflammatory epidermal cysts, pilomatricomas, and palmoplantar fibromatoses showed vascular signals (35). Given that benign lesions also showed vascularity, this feature must be used in conjunction with other parameters of ultrasound and correlated with clinical examination when assessing a suspicious lesion.

#### Can We Measure the Stiffness of Lesions With Ultrasound?

Real-time tissue elastography is a technique in which compression is applied to the tissues and stiffer (less deformed) tissues are demarcated from those that are less stiff (more deformed) (36). On ultrasound, the tumor elasticity is displayed using a color scheme where flexible tissues appear red, medium elasticity as green and yellow, and stiff tissues as blue (36). The strain ratio can also be calculated, which is a measure of the elasticity of the tumor relative to that of the neighboring tissue (36).

In an assessment by Botar-Jid et al. (36) of a group of patients with cutaneous melanoma scanned with tissue elastography, it was found that melanoma elasticity was a significant descriptor as 45.24% of the tumors were stiff and 42.86% had moderate elasticity (36). The strain ratio comparing the melanomas and the hypodermis as well as the melanomas and the dermis showed a strong relationship (36). This group also previously reported thicker tumors had lower elasticity, while thinner ones were more elastic (37). A similar study used real-time elastography for superficial spreading melanoma and found predominance of dark blue regions (rigid) within the melanomas compared to green areas in benign nevi (moderate elasticity) (38).

The strain ratio has been quantitatively evaluated in using elastography for various cutaneous carcinomas. It was found in a cohort of 55 patients with a total of 67 lesions (29 malignant and 38 benign) that all the malignant lesions had a strain ratio greater than or equal to 3.9 and all benign lesions had a strain ratio less than 3.0 (39). The malignant lesions in their study were basal and SCCs. Dasgeb et al. proposes that the malignant tissues have a higher strain ratio and are less compressible not only due to the nature of malignancy but also because of increased extracellular matrix deposition and greater adherence to surrounding tissues (39).

At this point in time, initial research findings of tissue elastography for skin cancers show promise but additional studies with larger sample sizes across multiple centers are still needed to evaluate the clinical implications of elastography (39). Further research also requires standardization and testing of protocol reliability in terms of how strain ratios are calculated, ultrasound gel pads used, and lesions that are assessed (40).

Moreover, recent studies have discussed the use of acoustic radiation force impulse (ARFI) for the assessment of skin conditions. ARFI is a method of elasticity measurement performed without using the transducer to physically compress tissue, but instead generating acoustic radiation force in tissue and monitoring the tissue displacement (41). Studies have assessed the feasibility of using ARFI and shear wave elasticity imaging (SWEI) for cutaneous sclerotic diseases such as systemic sclerosis (SSc). In one study, the modified Rodman skin score for diffuse cutaneous SSc was compared with the shear wave velocity (SWV) of the tissues (42). The shear wave elasticity values were higher in patients with SSc in the right and left dorsum of the hands, right and left forearms, and right and left dorsum of the feet. The SWV increased with increasing skin scores except three (42). The displacement of tissue is lower in sclerotic skin and the shear wave speed higher compared with normal skin. This is in keeping with what we know that in sclerotic skin manifests as being stiffer (43). Ultrasound elastography has the potential to provide an assessment of disease progression as the extent of external skin involvement is a marker of overall outcomes (40). Similar to strain imaging, further research is needed to assess protocol reliability given that SWEI is sensitive to the degree of compression of the tissue and would need testing with and without gel and on different body surfaces (40).

The usefulness of sonoelastography in the assessment of psoriasis remains equivocal. An ongoing study of patients with plaque psoriasis under topical corticosteroid treatment by Cucoş et al. (44) yielded that conventional B-mode ultrasound is useful for visualizing three layers: a superficial highly echogenic layer, an intermediate layer which is hypoechoic or anechoic, and a deeper layer with intermediate echogenicity. The intermediate band thickness, the region from the entrance echo to the dermis (and papillary dermis), is the most important sonographic parameter for determining response to treatment (44). However, in the study, there were no significant changes of dermal elasticity with corticosteroid treatment (44).

#### Integrative Analysis for Assessing Benign Versus Malignant Lesions

The use of a multi-modal ultrasonographic approach to identifying malignant tumors is beginning to take shape. A study published in 2014 by Crisan et al. evaluated 23 patients with clinical exam, dermoscopy, HFUS (20 MHz), conventional US, contrast elastography, and CEUS. Ultrasound examination correctly identified *all* the malignant tumors as hypoechoic on gray-scale US in keeping with histological findings. The MST had a blood flow rate greater than 2 cm/s on Doppler, while all BST had a blood flow rate less than 2 cm/s (31). Moreover, vessel distribution differed between malignant and benign tumors—the Doppler signal was found in 16 of 18 MST and 12 of 18 had central vasculature only (seen only in MST) and the circulatory pattern was disorganized, while all 5 BST had peripheral vasculature and were relatively organized. Finally, the contrast wash-out time was significantly higher in MST (31).

It is apparent that numerous studies have been conducted using various ultrasound techniques to characterize benign and malignant cutaneous lesions. Skin tumors whether benign or malignant are often hypoechoic on ultrasound (45). However, there are some distinguishing features such as BCCs can have hyperechoic spots within the lesion from either cysts or microcalcifications (45). Table 2 summarizes the characteristics that have been reported for each type of skin lesion given different parameters on ultrasound.

**Table 2:** Benign and malignant dermatologic lesion findings with multimodal ultrasonography.

Type of lesion	Echogenicity and homogeneity	Dimensions/shape	Doppler assessment	Contrast enhancement	Elastography
Basal cell carcinoma (31)	Hypoechoic and inhomogenous; may have hyperechoic or anechoic spots (24) (ultrasound can be used to identify growth patterns) (10)	Deep tumor borders visualized with 13–15 MHz	Uneven vascularization, central/mixed circulatory model, 1–2 supply vessels, >2 cm/s	Inhomogenous contrast load, wash out time rapid	Increased rigidity (strain ratio ≥3.9) (39)
Squamous cell carcinoma (SCC)	Hypoechoic and inhomogenous (46)	Irregular contours (46)	Mixed circulatory model, higher Vp and Vd, and lower RI and PI (32)	Utility in detecting metastatic nodes in head and neck SCC (47)	Increased rigidity (strain ratio ≥3.9) (39)
Melanoma	Hypoechoic and homogenous (25), oval or spindle shape (48) (*superficial spreading melanoma is very thin*)	Breslow thickness more accurately classified with higher frequencies ex. 75 MHz (23)	Vascular signals may appear later in time	Described as a tool to measure tumor response to antiangiogenic treatments and/or for detection of lymph nodes (49, 50)	Rigid to moderate elasticity (38)
Benign lesions (31)	Hypoechoic or anechoic and inhomogenous	Oval or spindle shape	More peripheral circulatory model or mixed, <2 cm/s	Slow wash out time, Weak and uneven loading of contrast	Moderate elasticity (strain ratio <3.0) (39)

*PI, pulsatility index, RI, resistant index; Vp, peak velocity*.

There are some limitations to the use of ultrasound in assessment of cutaneous lesions. Compared to histology, the tumor thickness can be overestimated with ultrasound due to perilesional infiltrates, retraction of tumor if extracted for histology, and with variations in ultrasound operator (51). Furthermore, it does not differentiate between tumor border and adjacent inflammation (10). As mentioned above, vascularity is seen in a wide variety of benign tumors such as angiomas, and thus correlation with clinical examination and other imaging features is needed (33).

### Can Ultrasound Be Used for Diagnosis of and Evaluating Extent of HS?

Hidradenitis suppurativa, previously known as Verneuil disease, is a chronic inflammatory condition that causes painful, suppurating, and often malodorous lesions that arise from the pilosebaceous unit in apocrine gland-rich locations such as the axilla, inguinal, and perianal regions (52, 53). Hair follicles become plugged secondary to hyperkeratosis, and the pilosebaceous units become dilated to the extent that they rupture into the nearby dermis. This can lead to abscess formation and infections, and when the follicles become re-epithelialized, sinus tracts can form in which bacteria become trapped (54).

In practice at the moment, the analysis of lesions is performed using the Hurley classification or Sartorius score which heavily relies on counting lesions with the naked eye (55). In the recent two decades, several researchers have explored the use of imaging technologies such as mammography, MRI, and ultrasound to evaluate the extent of the disease. This section validates the use of ultrasound a primary diagnostic tool for HS and its usefulness in both pre- and post-operative assessment.

#### Preliminary Reports of HS With Ultrasound

One of the first studies that used gray-scale ultrasound to examine the hair follicles in HS compared with healthy controls was in 1997 (56). The authors depicted lesions to be rounded, hypoechoic structures that extended into the subcutaneous tissue. Hypoechoic bands were present subepidermally; deeper in the dermis there were alternating bands of hyper- and hypoechogenicity (56). The authors found significantly increased skin thickness and follicle area in the axilla, and significantly increased skin thickness and follicle shape (superficial:deep diameter) in the genitofemoral region compared with healthy controls (56).

Another study comparing seven HS patients with eight healthy controls using 15.7 and 17.5 MHz linear ultrasound probes showed HS lesions to be dermal fluid collections with varying sizes (57). The average dermal thickness in HS patients was 3.3 mm (with a lower echogenicity) and 1.4 mm in healthy controls. In HS patients, hair follicles in the non-affected skin were deemed to be enlarged (57). While the lesions identified on ultrasound correlated with those clinically identified, on ultrasound *all* of the lesions extended beyond the clinically demarcated borders (57). Other studies have confirmed that clinically the disease extent is underestimated compared with ultrasound (58). Lesions are more widespread than the borders that are marked on physical exam, which helps in both preoperative planning and in detection of subclinical lesions.

#### Recent Literature Evaluating HS Features With Ultrasound

In the recent literature, further features of HS lesions have been depicted on ultrasound. In 2010, Kelekis et al. executed a prospective study of 19 patients with a 7–12 MHz ultrasound. This group found significant differences in thickness of epidermis and dermis assessed in the groin, buttocks, pubis, inframammary region, axilla, and back (59). The ability to see a discontinuity of the dermal–hypodermal junction indicated potential spread of the circumscribed lesions. This was the first paper that correlated the Hurley classification score with ultrasound scoring based on size, resistance index on spectral blood flow analysis, and epidermal–dermal boundary break (59). Newman et al. in 2013 presented two cases of HS patients who were scanned with mammography as well as gray-scale ultrasound and spectral blood flow analysis in real-time. Intradermal, hypoechoic lesions (some with central echogenicity) were shown to also have peripheral vascularity. Vascularity and resistance index can be other identifying features of this methodology in examining HS lesions (54).

One of the most promising studies was recently published in 2017 by the American Society for Dermatologic Surgery in which a group called DERMUS, composed of physicians working with HS patients, was evaluated to put together a consensus ultrasound report on HS (53). Ninety-three percent of the experts concluded that the HS final report should include all the three lesion subtypes, which are pseudocysts, fluid collections, and fistulous tracts. Other parameters such as the connection of fistulous tracts and hair tracts within the fluid collections and fistulous tracts were agreed upon by 86 and 79% of experts who responded (53). Eighty-six percent considered it important to measure major axes and thickness of each lesion, and 100% of experts recommended lymph node presence and color Doppler to be reported (53). Physical examination only allows visualization of large, inflamed, erythematous lesions rather than those that are subclinical. Clinical examination does not allow for differentiation between types of lesion, thus the creation of such a standardized ultrasound report can help remove barriers to staging HS (53).

**Figure 2:**
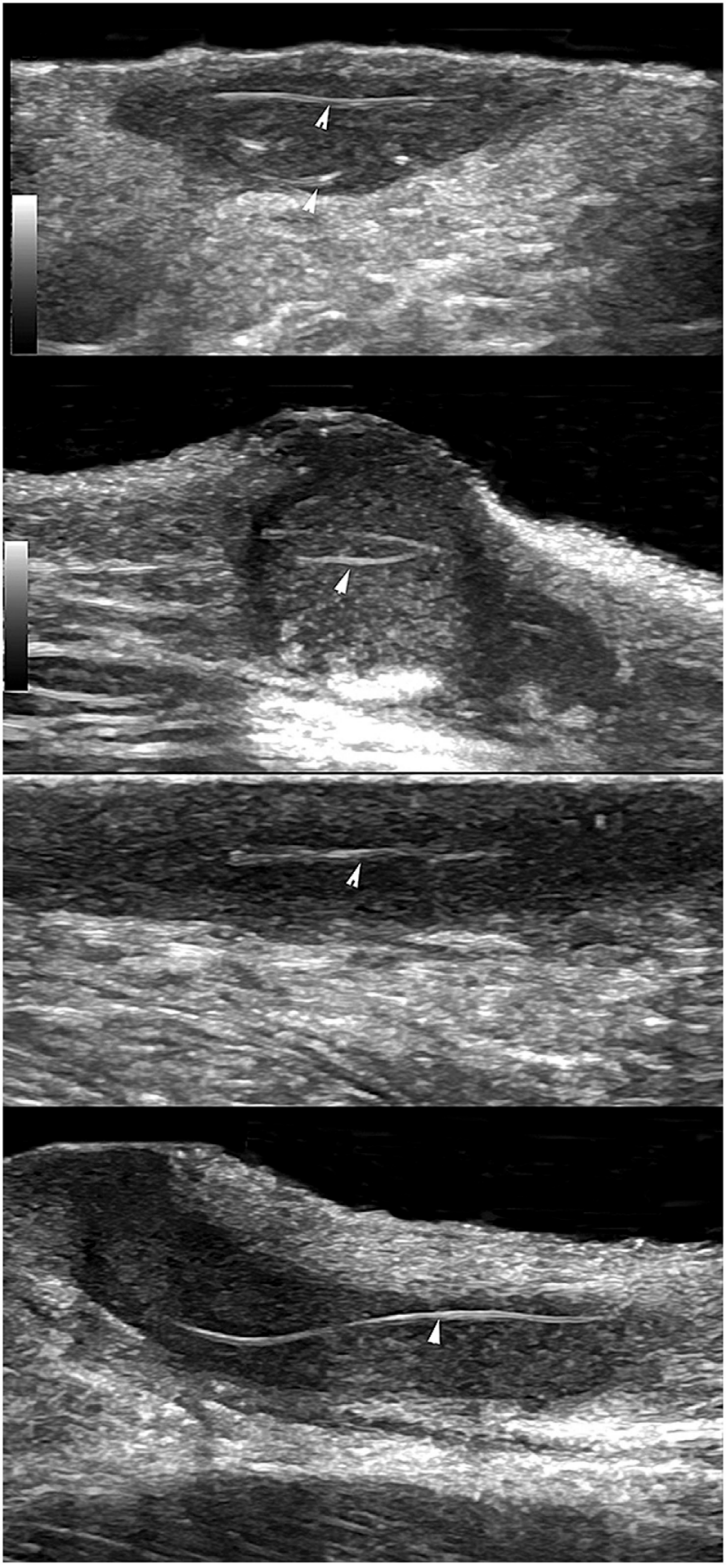
Using ultrasound to detect retained hair tracts (indicated by the arrows) within fistulae in hidradenitis suppurativa. This is indicative of regions that could flare with further inflammatory reactions and worsen extent of the disease. The mean length of hair tracts was 4.4 mm with a wide dispersion (SD of 6.9 mm) and a range of 2–31.7 mm. Images were taken with a General Electric Logic E9 with a maximum ultrasound bandwidth of 18 MHz. Permissions obtained to reprint from Wortsman and Wortsman (61).

In patients with HS, recurrences of lesions are very common even after surgical excision and reconstruction. A study of 20 patients compared their own assessment of their flare-ups and investigator analysis of ultrasound. Linear probes of variable frequencies (6–18 and 10–22 MHz) were utilized and an association was shown with the diameter of nodules and patient assessment of flare-up (60). Another predictor of recurrence may be abnormalities in hair tracts. For example, a study in 2015 reviewed the sonographic images of 50 HS patients and 40 patients (80%), which showed ectopic hair fragments in the fistulae or fluid collections within the dermis or hypodermis (61). This can be seen in Figure 2—there were abnormalities in hair fragment size and some hair tracts were over 3 cm in length (61). The ability to detect this could indicate regions that may flare with further inflammatory reactions and worsen the disease. Ultrasound can be very useful as a rapid tool for assessment in patients postoperatively to monitor disease stability and risk factors that are affecting regrowth of fistulous tracts.

This analysis has been replicated in the pediatric population to better understand HS in children. A retrospective analysis of 12 children with HS used color Doppler ultrasound to determine the number of fluid collections, fistulas, hair tracts, pseudocysts, and HS staging (62). Anatomic involvement in children with HS seems to follow the same ultrasound pattern as is observed in the adult population (62). Ultrasound showed the presence of subclinical lesions that were not reliably identified using palpation. Similar to the way that color Doppler has been used in the adult population to assess frequency of fistulous tracts and to stage lesions, the potential for this modality to do the same for children has merit from both a diagnostic and treatment perspective.

#### Limitations to the Use of Ultrasound for HS

The use of ultrasound in the recent years has rapidly changed our ability to view subclinical lesions in HS as well as understand the extent and boundaries of clinically visible lesions. The compatibility of ultrasound findings with the Hurley scoring system in both the adult and pediatric populations is very valuable in transitioning this into a bedside tool for diagnostic and perioperative purposes.

Despite this, some limitations exist. Ultrasound findings may support diagnoses for other cutaneous diseases. The findings seen may not allow for clear delineation between HS lesions, acne, folliculitis, or cutaneous nodules (63). In addition, transperineal ultrasound and anal ultrasonography are useful tools to separate HS fistulas from rectal fistulas or abscesses (64). However, MRI may be needed for evaluating extensive anogenital and deep lesions (63). Moreover, HS lesions generally need to be larger than 0.1 mm to visualize them on ultrasound, which may impact initial detection (60).

## IR-Based Imaging Modalities

The IR light spectrum uses wavelengths from 0.7 to 1,000 μm, longer than of visible light, to visualize structures that are deep to the skin. The degree to which the light penetrates is dependent on the interaction of the light with molecules such as water and hemoglobin (65). In this section, modalities such as RCM, OCT, and SIAscopy are discussed and Table 3 summarizes the sensitivities and specificities reported for these modalities.

**Table 3:** Receiver operating characteristics for infrared-based imaging modalities in identifying malignant lesions compared with histopathology.

	Level of evidence	Sensitivity	Specificity	Differentiating groups
Reflectance confocal microscopy (66)	Prospective primary research article (*n* = 1,279 lesions)	95.3%	83.9%	Skin cancers from benign lesions
Optical coherence tomography (67)	Primary research article (*n* = 104 patients)	79–94% (depending on experience)	85–96% (depending on experience)	Non-melanoma skin cancers from benign lesions and normal skin
Spectrophotometric intracutaneous analysis (7)	Prospective primary research article (*n* = 188 lesions)	83.9%	46.1%	Skin cancers from benign lesions

### Reflectance Confocal Microscopy

Over the past 5 years, RCM has been acknowledged as a tool to assess skin *in vivo*. This section highlights studies that have discussed RCM for classifying melanomas and mapping them before biopsy, as well as other skin carcinomas and monitoring their response to therapy.

#### RCM in Melanomas

One of the primary uses of RCM is to visualize melanomas. A study in 2015 by Stanganelli et al. articulated that the features on RCM including atypical cells and architectural disarray were included as those that were correlated with actual diagnosis of melanoma (68). In their study, the RCM features of melanoma also occurred often in benign lesions and this highlights the importance of having algorithms to delineate between nevi and melanoma (68). RCM can increase the accuracy of melanoma diagnosis if used in addition to standard dermoscopy such as in cases where there are minor or moderate changes in dermoscopy and a positive RCM score would sway one toward a biopsy (68). A systematic review of the diagnostic accuracy of RCM for melanoma diagnosis when lesions are clinically equivocal yielded a per lesion sensitivity of 93% and a specificity of 76% (69). This reinforces the utility of RCM as an add-on tool to dermoscopy; however, the downsides to this are that false positives that were already declared as “positive” by a pre-existing algorithm will remain misdiagnosed. Furthermore, the lesions that are true positives but declared as “negative” by RCM may be missed melanomas (69).

**Figure 3:**
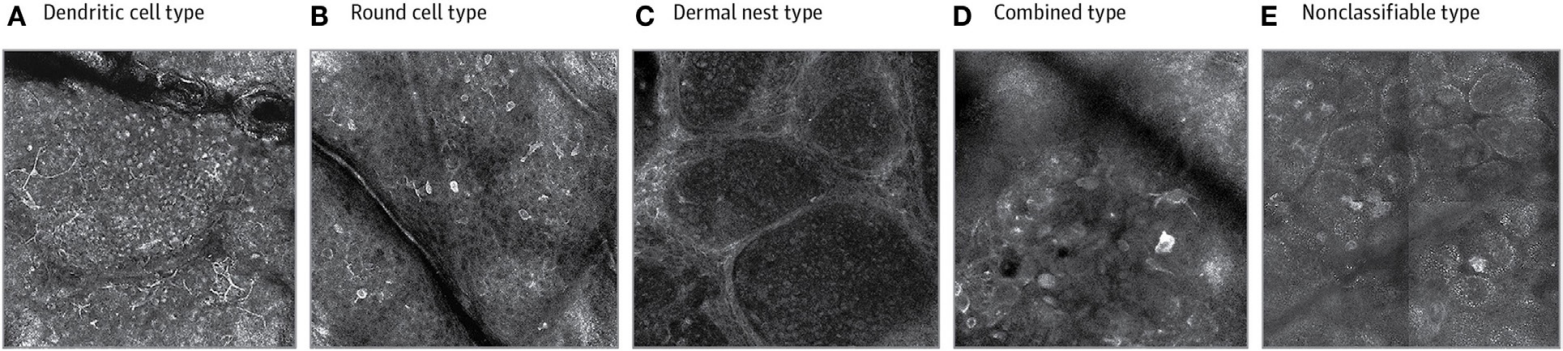
Reflectance confocal microscopy has allowed for sub-classification of melanomas using morphological descriptions. Permissions obtained to reprint from Grazziotin et al. (70). In their study, it was found that dendritic cell melanomas **(A)** were linked to more sun exposure and round cell melanomas **(B)** occurred in patients with familial melanomas and fairer skin. Dermal nest **(C)** and combined **(D)** melanomas were associated with a lack of pigmented network on dermoscopy and thicker tumours on histology. The morphological expression can also be linked to tumor behavior, for example, the non-classifiable type **(E)** had less atypia on basal layer melanocytes and had lower asymmetry, border, color, dermoscopic structures (ABCD rule in dermoscopy) scores using dermoscopy.

In terms of melanoma sub-classification, a recent classification system has been developed for melanomas based on RCM (70). The classification groups are dendritic cell, round cell, dermal nest, combined-type, and non-classifiable melanomas. These classes have been associated in the study with various clinical characteristics as per a cross-sectional retrospective study by Grazziotin et al. in 2016 in patients with a history of multiple melanomas or hereditary familial melanoma (see Figure 3). For example, they found that dendritic cell melanomas were typically found in older patients, in cases with more intense sun exposure, and were significantly associated with lentigo maligna (LM) subtype (70). These dendritic cell tumors grow more slowly in comparison to round cell tumors which aggregate in clusters and can move into the dermis more rapidly. Round cell melanomas were in fairer skinned patients and those with a positive family history (70). Given these relationships between morphological features of melanomas under RCM and other clinical characteristics of the phenotype and histological subtype, clinicians can gain a sense of tumor behavior from their RCM features (70).

**Figure 4:**
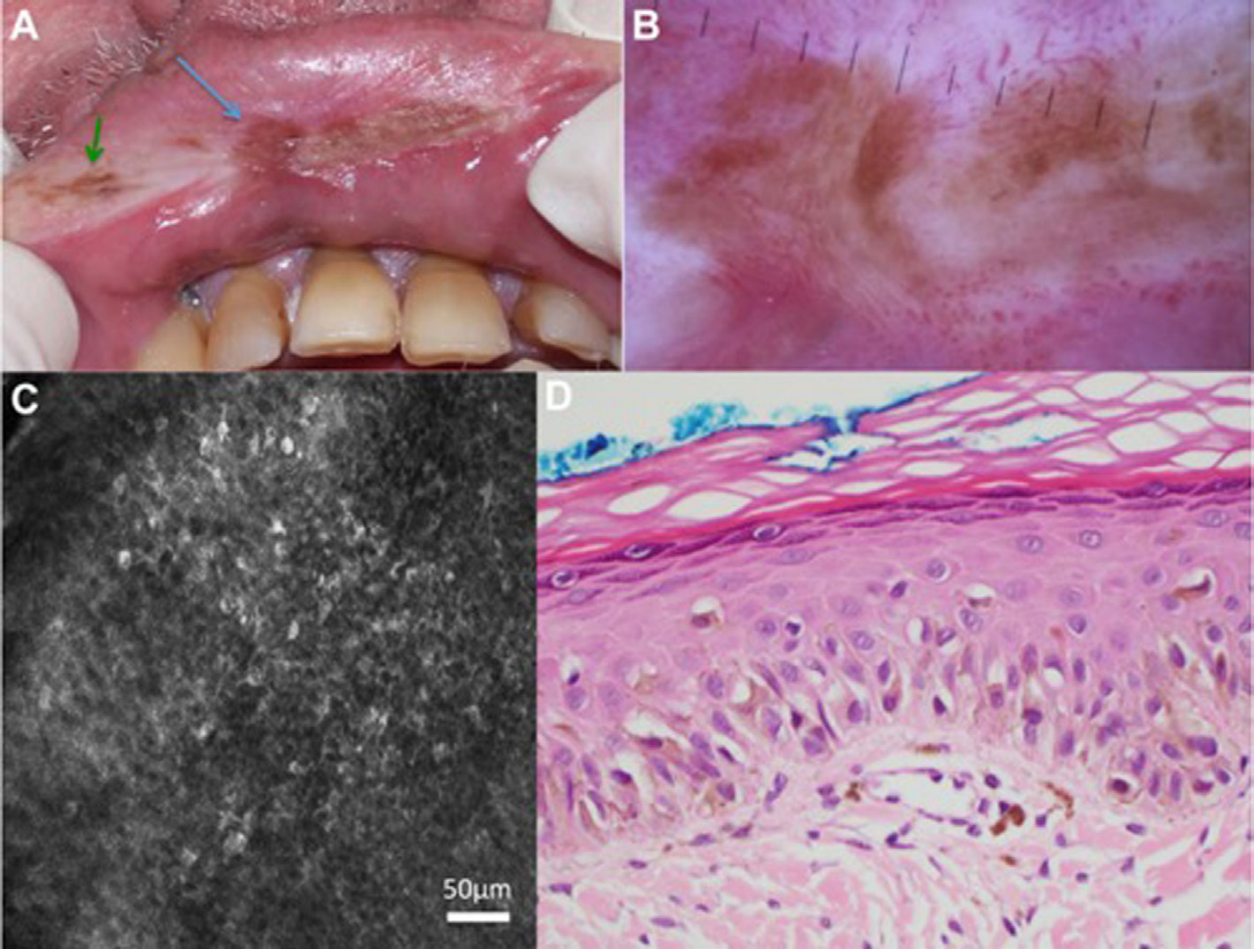
Patient with previously treated lentigo maligna (LM) melanoma had a new brown pigmented areas (indicated with the arrows) on clinical examination **(A)** and dermoscopy **(B)**. Reflectance confocal microscopy (RCM) of the pigmented area in the middle of the upper lip (blue arrow) showed round cells with dendritic processes in the epidermis **(C)**. RCM-targeted biopsy of this was performed and histopathology confirmed recurrence of LM **(D)**. Not shown in this figure: the pigmented area indicated by the green arrow showed features in keeping with solar lentigo on RCM. Permissions obtained to reprint from Maher et al. (71).

Reflectance confocal microscopy has also been useful in guiding diagnosis and treatment of LM melanomas and can also be used to guide and monitor Mohs microsurgery (15). In 2017, a prospective study was published by Maher et al. with eight patients who underwent RCM-guided biopsies of lip hyperpigmentation, two of which were *in situ* oral melanoma recurrences. See Figure 4 for the RCM images which demonstrate how the lesion was mapped before biopsy (71). It is noteworthy that morphology differs based on stage and behavior and the combination of dermoscopy and RCM can help to capture this.

#### What Are the Other Uses of RCM?

Recent literature by Borsari et al. (66) evaluated skin lesions that were referred for RCM because of at least one prior evaluation that was clinically and/or dermoscopically equivocal (66). There were 1,279 lesions assessed with RCM and compared with histopathology, this yielded a sensitivity of 95.3% and a specificity of 83.9% in accurately diagnosing the skin cancers (including BCC, SCC, and melanomas) (66). From the statistical analysis in this study, favorable indications for the use of RCM included localization to the head and neck because of the ease of exploration with RCM. Other favorable indications included the presence of sun-damaged skin (as RCM evaluates flat lesions well), observed regression on dermoscopy, and BCC-dermoscopic criteria (66). RCM performance significantly correlated with lesion diameters greater than 6 mm and erosions in the lesions (66). RCM was negatively correlated with peripheral streaks (often signifying Spitz or Reed Nevi) and therefore one may not need a RCM assessment if these are seen (66).

Reflectance confocal microscopy has also been shown to be a measurement tool for response to therapy. According to Xiang et al. (72), the features on RCM of invasive SCC are the atypical honeycomb pattern composed of keratinocytes of differing shapes and sizes at the spinous-granular level, the absence of a cobblestone pattern, and the presence of non-edged dermal papillae (72). Interestingly, these are also the features that are helpful when assessing treatment response after therapy.

Analysis *in vivo* of BCCs using RCM helps us to confirm complete response after surgical excision and assess for residual tumor. In a study of seven patients with nine locally advanced BCC’s, two cases developed keratinocytic neoplastic proliferations post-treatment at the tumor site consistent with basal cell features and another developed actinoytic tumors at a distal site and these were identified with RCM and treated subsequently (73). While the diagnostic accuracy will vary with assessor capabilities, RCM is a useful tool when it is challenging to examine pigmented lesions with a dermatoscope. RCM is a helpful add-on to dermoscopy to increase the diagnostic sensitivity and aid in the clinical decision making around referring to surgery and it allows clinicians to avoid biopsying lesions unnecessarily (74–76).

A pilot study in 2010 by Astner et al. used RCM to examine various vascular lesions including benign vascular malformations (spider angioma), acquired benign vascular tumors (cherry angioma), and benign congenital malformations (angiokeratoma). The RCM features of the vessels in these benign lesions were described in terms of their diameter, tortuosity, and flow velocity (77). For example, RCM has the ability to differentiate between the large-diameter venules in a port wine stain and the smaller vessels in benign capillary proliferations (77). Moreover, pyogenic granuloma would show a large number of tortuous and dilated capillaries but to separate this from melanoma, other RCM characteristics of melanoma would be present in the latter case (77). The vascularity of lesions can be depicted with RCM, but this must be taken into account with other confocal microscopic features and the clinical picture.

The diagnosis and exclusion of dermatophytic infections has been benefited by RCM. Friedman et al. (78) had a 100% sensitivity in reaching diagnoses of dermatophytic infections or assessing response to antifungal treatment when comparing RCM with either KOH prep, fungal culture, or skin biopsy. The presence of hyphae is the feature that one would look for on RCM (78).

### Optical Coherence Tomography

The IR spectrum is subclassified into near-IR, mid-IR, and far-IR based on wavelength. OCT is a technique that uses non-ionizing, near-IR light (65). OCT first entered the realm of skin imaging in 1997 (79). This section gives a brief overview of the recent advances in OCT for skin imaging.

A comparative study of presurgical skin infiltration depth measurements using HFUS and OCT of melanocytic lesions showed similar axial resolution. However, OCT was slightly more precise in terms of thickness determination and HFUS had better contrast (80). Moreover, the researchers found that these OCT and HFUS were comparable to histopathology in the way that they estimate infiltration depth of melanomas or melanocytic nevi *in vivo* (80).

Optical coherence tomography has strong diagnostic accuracy for superficial BCC’s because of its ability to measure depth in tumors that are less than 0.4 mm (81). This has been corroborated by a study performed by Ulrich et al. in 2015. Literature has shown that when conventional OCT and dermoscopy are added to clinical examination in assessing BCC’s, this increases the sensitivity of recognizing the BCC from 90.0 to 95.7% and the specificity from 28.6 to 75.3% (82). In a study of 104 patients, when OCT was used to assess non-melanoma skin cancer (NMSC) from benign lesions and normal skin, sensitivity was 79–94% and specificity was 85–96% (67).

**Figure 5:**
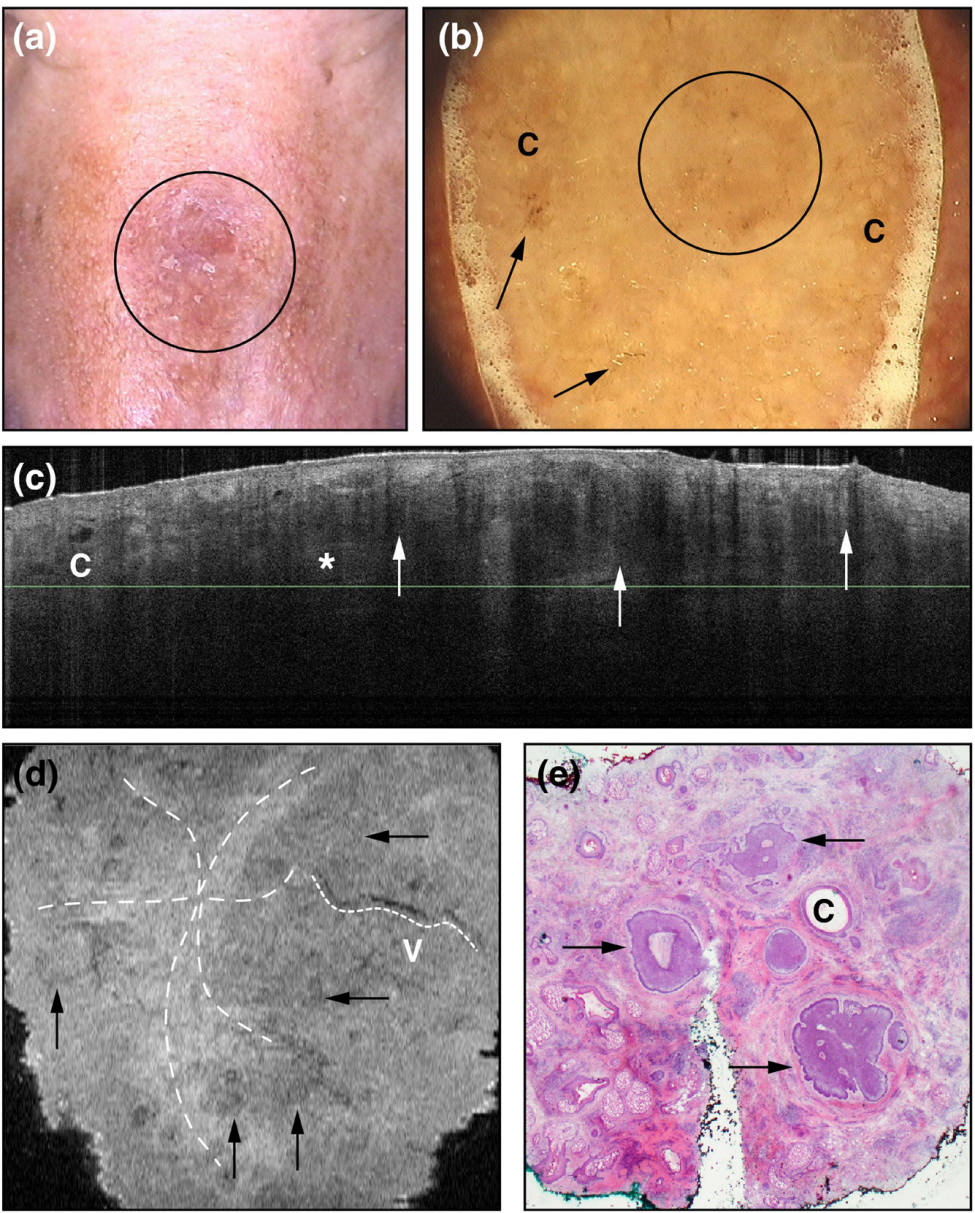
A demonstration of sclerosing basal cell carcinoma on the nose (circle) visualized with **(A)** clinical examination and **(B)** dermoscopy (“c” representing milia-like structures and the arrows pointing to erosions). Slide-mode optical coherence tomography is depicted in box **(C)** with nodules that are hyporeflective with hyporeflective rims (arrows) and hyperreflective peritumoral borders (asterisk) and a cyst marked “c.” In box **(D)**, the en-face mode gives information on the peritumoural fibers (star-shaped dotted line), vessels (marked “v” with smaller dotted line), and hyporeflective nodules with bright centers (arrows). Histopathology in panel **(E)** demonstrates peritumoural fibers surrounding tumors (arrows) and cysts marked with “c.” Permissions obtained to reprint from von Braunmühl et al. (1).

Previously, cross-sectional slices (vertical) were only obtainable; however, recently, an en-face (horizontal) mode of OCT has been used and this has allowed for more defining features of BCC’s to be identified (1). With the combination of cross-sectional and en-face mode, BCCs on OCT were confirmed to be lobulated/ovoid in structure with a hypoechoic peritumoural rim and hyperechoic peritumoural stroma, have branching vessels, and have compressed fibrous bundles between lobules (1). Figure 5 demonstrates a sclerosing BCC visualized using clinical observation, dermoscopy, slice mode OCT, en-face mode OCT, and histopathology.

Other forms of OCT include HD-OCT and dynamic optical coherence tomography (D-OCT). HD-OCT has improved lateral resolution and therefore offers further information about structure and cellular detail while forgoing the penetration depth of conventional OCT (17). It has been shown that HD-OCT is particularly useful in cosmetically sensitive areas such as the face or scalp (2). However, the performance of HD-OCT depends on tumor thickness and one study by Gambichler et al. in 2015 demonstrated that HD-OCT has a high false-negative rate in thin melanomas and a high false-positive rate in dysplastic nevi (83).

Dynamic optical coherence tomography also known as speckle-variance OCT has the ability to display the vascular architecture within skin lesions (84). Schuh et al. demonstrated blood flow patterns within lesions using a dynamic OCT algorithm in a device called VivoSight (84). The researchers commented on the ability of D-OCT to differentiate between actinic keratoses (AKs), Bowen’s disease (SCC *in situ*), and invasive SCC. AKs were reticular-appearing, Bowen’s disease displayed grainy patterns, and invasive SCCs appeared irregular with changing vessel diameters (84). D-OCT displays the vascular patterns of lesions which is an asset to any tool when assessing malignant potential.

A systematic review of the OCT in dermatology literature by Olsen et al. in 2015 yielded that most of the OCT studies were in NMSC and the diagnostic accuracy of this was shown to be 87.4% (85). There exists some literature of OCT for melanoma as described above, but this is limited by smaller sample sizes of malignant lesions (85). OCT studies focus on epidermal thickness and morphological changes to ultraviolet radiation and using this modality in combination with others would increase its applicability to the clinical setting (85).

### What About SIAscopy?

Spectrophotometric intracutaneous analysis is a technique that covers the visible spectrum and a portion of the IR spectrum to visualize pigmented lesions (86). The SIAscope relies on the reflectance spectrum created by chromophores in the skin and displays an image after collecting information on the quantity and location of these chromophores within the epidermis and papillary dermis (7).

Dermoscopy is a technique that lends itself to subjective interpretation by the clinician and requires time to become adept at using. SIAscopy has been implicated as a useful point-of-care tool for delineating when to be concerned about a pigmented lesion and can aid general practitioners in deciding when to refer for further work-up (7), Emery et al. (9) developed a scoring system validated on UK and Australian populations, known as the primary care scoring algorithm, in which dermal melanin, blood vessels, blood displacement, diameter, and age increased suspiciousness of the lesion. The algorithm has criteria to help rule out benign seborrheic keratosis and benign hemangiomas when aiming to identify melanomas (9).

Using this algorithm described above, Sgouros et al. (7) examined 188 lesions *via* SIAscopy. In comparison to dermoscopy, SIAscopy had a sensitivity of 85.7% and specificity of 65.4% (7). 44 lesions were excised and 31 of these were malignant tumors; the sensitivity of SIAscopy was 83.9% in comparison to histopathology (7). 7 of the 13 benign excised lesions were scored as malignant by SIAscopy (7). In their study, 9 of 10 BCCs and 2 of 3 SCCs were classified as suspicious by SIAscopy scoring (7). Given these findings, authors have concluded that although SIAscopy cannot be used as a solely diagnostic tool, it represents an additional tool that can be used when deciding when to refer to a dermatologist for a suspicious pigmented lesion (7).

## Electrical Impedance Spectroscopy

Electrical impedance spectroscopy measures resistance of tissues by applying an alternating current using frequencies between 1 kHz and 2.5 MHz (4, 20). The shape, size, and membrane of cells can affect the resistance detected by EIS (4). The EIS system results in both a score (from 0 to 10) and a dichotomous response (negative or positive) using a given cutoff value (87). EIS has been specifically evaluated as a method for identifying malignant melanocytic lesions (8). Despite the various algorithms that exist for scoring melanocytic lesions such as asymmetry, border, color, dermoscopic structures (rule in dermoscopy) (ABCD) or 7-point checklist, diagnostic challenges arise frequently in these scenarios.

Rocha et al. conducted an observational, prospective study in 2017 in Australia to assess whether the EIS device *Nevisense* was a useful adjunct to short-term sequential digital dermoscopy imaging (SDDI) when examining melanocytic lesions. SDDI is performed at two separate appointments that are 3 months apart and if a significant change is detected, this is an indication for excision (19). If a *Nevisense* score of greater than or equal to seven was computed, lesions were automatically excised, otherwise lesions were re-assessed in 3 months (19). The researchers found that the sensitivity for diagnosing melanoma was 100% with this device and the specificity was 69.5% (19). EIS may be added to SDDI to improve diagnostic accuracy, expedite excisions, and avoid repeat appointments and a delay of 3 months for suspicious lesions with higher impedance scores (19).

On the contrary, other researchers noted in a retrospective study examining 22 atypical melanocytic lesions using EIS in a modified algorithm that EIS may have actually caused for unnecessary excisions (8). The authors noted that out of the seven lesions that were excised, three were histopathologically benign (benign nevi) but excised because of increased EIS scores without any dermoscopic changes. Ceder et al. (8) also did not find a correlation between dermoscopic changes on SDDI and increased EIS scores.

Although it is argued that this technique is a safe device to aid in detection of melanomas, the literature is equivocal as to whether the benefits outweigh the risks in terms of introducing EIS as an aid to decision-making for atypical nevi.

## Discussion

This review suggests an approach to diagnosing and managing skin lesions based on different modalities and functions. While some lesions may still need to be confirmed with histology, the recent dermatology literature throughout the world suggests such a shift toward the use of non-invasive modalities in the diagnostic work-up. They have been shown to have utility as adjunctive tools in recognizing malignant lesions, vascular lesions, and more specifically, staging diseases such as HS.

**Figure 6:**
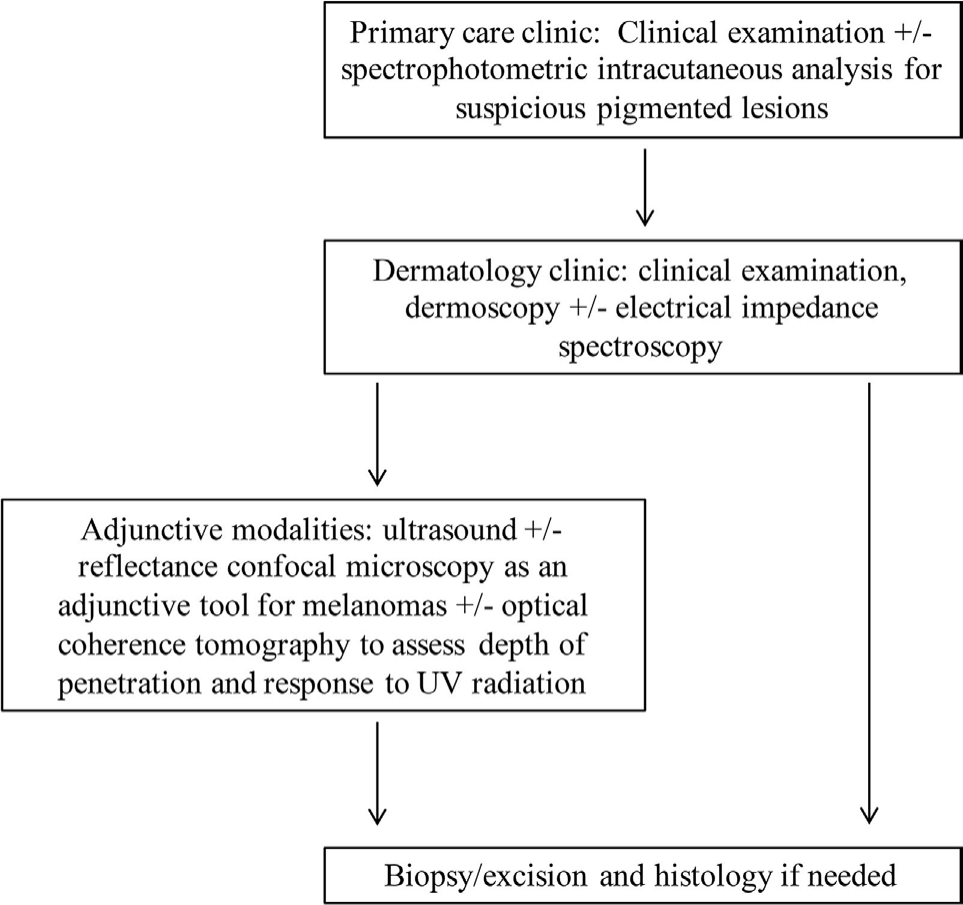
A proposal of a novel approach for examination of cutaneous diseases. Spectrophotometric intracutaneous analysis may be useful for primary care physicians in considering lesions before referral. Clinical visualization and dermoscopy are always the first component of the examination of a lesion. Some literature suggests that electrical impedance spectroscopy can aid in the decision-making process regarding excision. Ultrasound can be incorporated next with modification of frequency parameters to obtain optimal penetration depth; ultrasound features such as color Doppler for assessment of lesion vascularity can be used as needed. Reflectance confocal microscopy and optical coherence tomography may be helpful in specific cases, and biopsy and histopathology remain the final step if further diagnostic clarification is needed.

Figure 6 proposes an approach for suspicious skin lesions. Dermoscopy has significant inter-individual variability that is inherent to the technique. It has been proposed that in the primary care setting, physicians use SIAscopy to decide when to refer to dermatologists regarding suspicious lesions. The SIAscopy device has been approved by the United States Food and Drug Administration (FDA) and by Health Canada (6). In the dermatologist’s office, dermoscopy is used as a routine component of visits and has been described to be the gold standard for the clinical examination of pigmented skin lesions (88). Dermoscopy is not only more accurate than the naked eye in the diagnosis of cutaneous melanoma, for example, but has also been shown to be superior to SIAscopy in terms of specificity when assessing pigmented skin lesions (88, 89). Although EIS has been proposed at this stage, it is unclear whether the cost–benefit ratio favors this modality.

Some of the more expensive techniques include ultrasound (such as the VevoMD system by VisualSonics approved by Health Canada and FDA), RCM (such as the Vivascope^®^ approved by both the FDA and Health Canada), and OCT (such as VivoSight approved by the FDA but not yet for sale in Canada) (90–93). These may be used for specific cases in which further classification of a lesion is needed.

The techniques described above have a number of advantages and disadvantages (see Table 1). In terms of time to evaluation, RCM does require more time compared with the other modalities (on the order of minutes); however, it does have lateral resolution close to histopathology (2). Compared to RCM, OCT and ultrasound have improved depth penetration, with the depth penetration of ultrasound dependent on the frequency used. The downside of these techniques would be diminished lateral resolution (2).

Many of the modalities discussed rely on identifying features on the visualized images to classify lesions. With dermoscopy, there are various methods that can be adopted when evaluating melanocytic lesions. Examples of these include the ABCD rule, Menzies method, 7-point checklist, and the 3-point rule (89). In the Gambichler et al. study in 2015, they highlighted that from HD-OCT, users are provided with structural patterns and detail, and this underscores the importance for developing appropriate scoring systems to accurately capture which lesions are benign versus malignant (83). As articulated by Stanganelli et al., RCM features can be converted into an RCM score which can then be categorized as positive or negative (68). RCM has been recognized as an add-on to dermoscopy especially in cases where lesions are equivocal from dermoscopy or clinical examination and RCM can help sway clinicians when deciding whether or not to biopsy (68).

To address the importance of early detection of malignant lesions, one future direction is the use of deep learning algorithms in determining malignant potential. Esteva et al. reported on a deep convolutional neural network (CNN) which is a data-driven learning algorithm that is trained based on a set of images (including dermoscopic images) to recognize malignant lesions (94). The algorithm was tested for two cases: keratinocyte carcinomas versus benign seborrheic keratoses, and malignant melanomas versus benign nevi (94). The algorithm performed on par with 21 board-certified dermatologists on images that were confirmed with biopsy, indicating that this could have large clinical impact beyond a dermatology clinic setting (94).

Other future directions include the use of ultrasound to assess skin post-cosmetic procedures and to measure the degree of atrophy post-application of topical steroids (95, 96). Although not widely performed, literature has introduced the use of ultrasound measurement of regression of skin carcinomas and precancerous lesions post-photodynamic therapy and brachytherapy (97, 98). It is expected that this may be expanded and validated as a tool to measure tumor persistence or recurrence after other forms of nonsurgical treatment. This would be beneficial in a busy clinic setting to determine whether further treatment is necessary. It is also anticipated that research will also grow in the use of skin imaging for evaluation of collagenopathies (rather than biopsies) as these patients’ skin have a different response to physical impact and to guide treatment for bullous conditions (42, 99, 100).

## Author Contributions

The authors of this manuscript meet all of the criteria for authorship listed in the Frontiers author guidelines. SH contributed the search and synthesis of primary research articles. SF contributed to the technological elements of the text as well as made edits to the figures and tables. MB contributed sources within the field of dermatology for comparison within the text. NS is the principal investigator of this project and was involved in several steps of this project from idea generation to manuscript writing.

## Conflict of Interest Statement

The authors declare that the research was conducted in the absence of any commercial or financial relationships that could be construed as a potential conflict of interest. The reviewer CJ and handling Editor declared their shared affiliation.

## Abbreviations

ABCD, asymmetry, border, color, dermoscopic structures (rule in dermoscopy); AK, actinic keratosis; ARFI, acoustic radiation force impulse; BCC, basal cell carcinoma; BST, benign skin tumors; CEUS, contrast-enhanced ultrasound; D-OCT, dynamic optical coherence tomography; EIS, electrical impedance spectroscopy; HD-OCT, high-definition optical coherence tomography; HS, hidradenitis suppurativa; HFUS, high-frequency ultrasound; LM, lentigo maligna; mRSS, modified Rodman skin score; MST, malignant skin tumors; OCT, optical coherence tomography; RCM, reflectance confocal microscopy; PCSA, primary care scoring algorithm; PI, pulsatility index; RI, resistant index; SIAscopy, spectrophotometric intracutaneous analysis; SCC, squamous cell carcinoma; SSc, systemic sclerosis; SDDI, short-term sequential digital dermoscopy imaging; SWEI, shear wave elasticity imaging; SWV, shear wave velocity; TPUS, transperineal ultrasound; TTP, time to peak; Vm, mean velocity; Vp, peak velocity.

## References

[1] von Braunmühl T Hartmann D Tietze JK Cekovic D Kunte C Ruzicka T Morphologic features of basal cell carcinoma using the en-face mode in frequency domain optical coherence tomography. J Eur Acad Dermatol Venereol (2016) 30:1919–25. 10.1111/jdv.13704 27581090

[2] Cao T Tey HL High-definition optical coherence tomography – an aid to clinical practice and research in dermatology. J Dtsch Dermatol Ges (2015) 13:886–90. 10.1111/ddg.30_12768 26882379

[3] Tehrani H Walls J Price G Cotton S Sassoon EM Hall PN A prospective comparison of spectrophotometric intracutaneous analysis to clinical judgement in the diagnosis of nonmelanoma skin cancer. Ann Plast Surg (2007) 58:209–11. 10.1097/01.sap.0000235476.10517.bb 17245151

[4] March J Hand M Grossman D. Practical application of new technologies for melanoma diagnosis: part I. Noninvasive approaches. J Am Acad Dermatol (2015) 72:929–41. 10.1016/j.jaad.2015.02.1138 25980998

[5] MoleMate™SIMSYS™. About SIMSYS-MoleMate™. SIASscopy™: All Things MedX. (2017). Available from: http://www.medxhealth.com/Our-Products/SIAscopytrade;/overview.aspx (Accessed: December 9, 2017).

[6] Rigel D Farberg A Non-Invasive Technologies for the Diagnosis and Management of Skin Cancer, an Issue of Dermatologic Clinics. 1st ed (Vol. 35-4). Elsevier Health Sciences (ebook) (2017).

[7] Sgouros D Lallas A Julian Y Rigopoulos D Zalaudek I Longo C Assessment of SIAscopy in the triage of suspicious skin tumours. Skin Res Technol (2014) 20:440–4. 10.1111/srt.12138 24517201

[8] Ceder H Sjöholm Hylén A Wennberg Larkö A-M Paoli J. Evaluation of electrical impedance spectroscopy as an adjunct to dermoscopy in short-term monitoring of atypical melanocytic lesions. Dermatol Pract Concept (2016) 6:1–6. 10.5826/dpc.0604a01 27867738 PMC5108637

[9] Emery JD Hunter J Hall PN Watson AJ Moncrieff A Walter FM. Accuracy of SIAscopy for pigmented skin lesions encountered in primary care: development and validation of a new diagnostic algorithm. BMC Dermatol (2010) 10:9. 10.1186/1471-5945-10-9 20868511 PMC2954906

[10] Hernandez C del Boz J de Troya M Can high-frequency skin ultrasound be used for diagnosis and management of basal cell carcinoma? Actas Dermosifiliogr (2014) 105:107–11. 10.1016/j.ad.2013.09.004 24168914

[11] Knapik DA Starkoski B Pavlin CJ Foster FS. A 100–200 MHz ultrasound biomicroscope. IEEE Trans Ultrason Ferroelectr Freq Control (2000) 47:1540–9. 10.1109/58.883543 18238700

[12] NDT Resource Center. Characteristics of piezoelectric transducers. NDT Resour Cent (2012). Available from: https://www.nde-ed.org/EducationResources/CommunityCollege/Ultrasonics/EquipmentTrans/characteristicspt.htm (Accessed: September 4, 2017).

[13] Shung KK. High frequency ultrasonic imaging. J Med Ultrasound (2009) 17:25–30. 10.1016/S0929-6441(09)60012-6 20445825 PMC2863319

[14] Fink C Haenssle HA Non-invasive tools for the diagnosis of cutaneous melanoma. Skin Res Technol (2016) 23:261–71. 10.1111/srt.12350 27878858

[15] Rajadhyaksha M Marghoob A Rossi A Halpern AC Nehal KS Reflectance confocal microscopy of skin in vivo: from bench to bedside. Lasers Surg Med (2016) 49:7–19. 10.1002/lsm.22600 27785781 PMC5575825

[16] Calzavara-Pinton P Longo C Venturini M Sala R Pellacani G. Reflectance confocal microscopy for *in vivo* skin imaging. Photochem Photobiol (2008) 84:1421–30. 10.1111/j.1751-1097.2008.00443.x 19067964

[17] Boone MA Suppa M Dhaenens F Miyamoto M Marneffe A Jemec GB In vivo assessment of optical properties of melanocytic skin lesions and differentiation of melanoma from non-malignant lesions by high-definition optical coherence tomography. Arch Dermatol Res (2016) 308:7–20. 10.1007/s00403-015-1608-5 26563265 PMC4713458

[18] Claridge E Cotton S Moncrieff M Hall P Spectrophotometric intracutaneous imaging (SIAscopy): method and clinical applications. Chapter 37. In: Serup J Jemec GBE Grove G, editors. A Handbook of Non-Invasive Methods and the Skin. 2nd ed Boca Raton: CRC Press (2006). p. 315–25.

[19] Rocha L Menzies SW Lo S Avramidis M Khoury R Jackett L Analysis of an electrical impedance spectroscopy system in short-term digital dermoscopy imaging of melanocytic lesions. Br J Dermatol (2017) 177:1432–8. 10.1111/bjd.15595 28421597

[20] Welzel J Schuh S Noninvasive diagnosis in dermatology. J der Deutschen Dermatologischen Gesellschaft (2017) 15:999–1016. 10.1111/ddg.13347 28976087

[21] American Association of Family Physicians (AAFP). Skin biopsy site considerations: danger zones. Dermatol Proced (2014). Available from: http://www.aafp.org/test/fpcomp/FP-E_426/pt3-s4-s1.html (Accessed: September 4, 2017).

[22] Maj M Warszawik-Hendzel O Szymanska E Walecka I Rakowska A Antczak-Marczak M High frequency ultrasonography: a complementary diagnostic method in evaluation of primary cutaneous melanoma. G Ital Dermatol Venerol (2015) 150:595–601. 26333555

[23] Fernandez CI de Troya Martín M Fúnez Liébana R Rivas Ruiz F Blanco Eguren G Blásquez Sánchez N Preoperative 15-MHz ultrasound assessment of tumor thickness in malignant melanoma. Actas Dermosifiloq (2013) 104:227–31. 10.1016/j.ad.2012.06.007 22938997

[24] Jasaitiene D Valiukeviciene S Linkeviciute G Raisutis R Jasiuniene E Kazys R Principles of high-frequency ultrasonography for skin pathology. J Eur Acad Dermatol Venereol (2011) 25:375–82. 10.1111/j.1468-3083.2010.03837.x 20849441

[25] Guitera P Li LX Crotty K Fitzgerald P Mellenbergh R Pellacani G Melanoma histological Breslow thickness predicted by 75-MHz ultrasonography. Br J Dermatol (2008) 159:364–9. 10.1111/j.1365-2133.2008.08681.x 18565186

[26] Kučinskienė V Samulėnienė D Gineikienė A Raišutis R Kažys R Valiukevičienė S Pre-operative assessment of skin tumor thickness and structure using 14-MHz ultrasound. Medicina (Kaunas) (2014) 50:150–5. 10.1016/j.medici.2014.08.002 25323542

[27] Fujifilm VisualSonics Inc. Vevo MD: The World’s First Ultra High Frequency Ultrasound System for Clinical Use. (2017). Available from: https://www.visualsonics.com/product/imaging-systems/vevo-md (Accessed: August 5, 2017).

[28] University of Virginia. Emergency Ultrasound – Techniques – Transducer. (2013). Available from: https://www.med-ed.virginia.edu/courses/rad/edus/technique2.html (Accessed: April 10, 2018).

[29] Dybiec E Pietrzak A Adamczyk M Michalska-Jakubus M Wawrzycki B Lotti T High frequency ultrasonography of the skin and its role as an auxiliary tool in diagnosis of benign and malignant cutaneous tumors—a comparison of two clinical cases. Acta Dermatovenerol Croat (2015) 23:43–7. 25969912

[30] Stramare R Gazzola M Coran A Sommavilla M Beltrame V Gerardi M Contrast-enhanced ultrasound findings in soft-tissue lesions: preliminary results. J Ultrasound (2013) 16:21–7. 10.1007/s40477-013-0005-1 24046796 PMC3774904

[31] Crişan D Badea AF Crişan M Rastian I Gheuca Solovastru L Badea R Integrative analysis of cutaneous skin tumours using ultrasonographic criteria. Preliminary results. Med Ultrason (2014) 16:285–90. 25463879 10.11152/mu.201.3.2066.164.dcafb

[32] Petik B Yildiz H Differentiation of benign and malignant skin lesions with color and power Doppler ultrasonography. J Clin Anal Med (2013) 4:107–11. 10.4328/JCAM.938

[33] Scotto di Santolo M Sagnelli M Mancini M Scalvenzi M Delfino M Schonauer F High-resolution color-Doppler ultrasound for the study of skin growths. Arch Dermatol Res (2015) 307:559–66. 10.1007/s00403-015-1538-2 25604691

[34] Babcock DS Patriquin H LaFortune M Dauzat M. Power Doppler sonography: basic principles and clinical applications in children. Pediatr Radiol (1996) 26:109–15. 10.1007/BF01372087 8587808

[35] Yamaoka M Kuramochi A Takeuchi K Saitoh T Ikebuchi K Sonographic appearance of benign subcutaneous nodules – including color Doppler sonography. Rinsho Byori (2014) 62:432–9. 25051657

[36] Botar-Jid CM Cosgarea R Bolboaca SD Şenila S Lenghel M Rogojan L Assessment of cutaneous melanoma by use of very high frequency and real-time elastography. AJR Am J Roentgenol (2016) 206:699–704. 10.2214/AJR.15.15182 26866335

[37] Botar-Jid C Bolboaca SD Cosgarea R Şenila S Rogojan L Lenghel M Doppler ultrasound and strain elastography in the assessment of cutaneous melanoma: preliminary results. Med Ultrason (2015) 17:509–14. 10.11152/mu.2013.2066.174.dus 26649347

[38] Hinz T Winzel J Schmid-Wendter MH Real-time tissue elastography: a helpful tool in the diagnosis of cutaneous melanoma. J Am Acad Dermatol (2011) 65:424–6. 10.1016/j.jaad.2010.08.009 21763568

[39] Dasgeb B Morris MA Mehregan D Siegel EL. Quantified ultrasound elastography in the assessment of cutaneous carcinoma. Br J Radiol (2015) 88:20150344. 10.1259/bjr.20150344 26268142 PMC4730976

[40] DeJong HM Abbott S Zelesco M Kennedy BF Ziman MR Wood FM. The validity and reliability of using ultrasound elastography to measure cutaneous stiffness, a systematic review. Int J Burns Trauma (2017) 7:124–41. 29348976 PMC5768929

[41] Bruno C Minniti S Bucci A Mucelli RP ARFI: from basic principles to clinical applications in diffuse chronic disease – a review. Insights Imaging (2016) 7:735–46. 10.1007/s13244-016-0514-5 27553006 PMC5028343

[42] Hou Y Zhu QL Liu H Jiang YX Wang L Xu D A preliminary study of acoustic radiation force impulse quantification for the assessment of skin in diffuse cutaneous systemic sclerosis. J Rheumatol (2015) 42:449–55. 10.3899/jrheum.140873 25593239

[43] Lee SY Cardones AR Doherty J Nightingale K Palmeri M Preliminary results on the feasibility of using ARFI/SWEI to access cutaneous sclerotic diseases. Ultrasound Med Biol (2015) 41:2806–19. 10.1016/j.ultrasmedbio.2015.06.007 26259888 PMC4593719

[44] Cucoş M Crişan M Lenghel M Dudea M Croitoru R Dudea SM Conventional ultrasonography and sonoelastography in the assessment of plaque psoriasis under topical corticosteroid treatment – work in progress. Med Ultrason (2014) 16:107–13. 10.11152/mu.2013.2066.162.mc1mc2 24791841

[45] de Oliveira Barcaui E Carvalho AC Lopes FP Piñero-Maceira J Barcaui CB. High frequency ultrasound with color Doppler in dermatology. An Bras Dermatol (2016) 91:262–73. 10.1590/abd1806-4841.20164446 27438191 PMC4938268

[46] Mandava A Ravuri PB Konathan R. High-resolution ultrasound imaging of cutaneous lesions. Indian J Radiol Imaging (2013) 23:269–77. 10.4103/0971-3026.120272 24347861 PMC3843339

[47] Dudau C Hameed S Gibson D Muthu S Sandison A Eckersley RJ Can Contrast-enhanced ultrasound distinguish malignant from reactive lymph nodes in patients with head and neck cancers? Ultrasound Med Biol (2013) 40:747–54. 10.1016/j.ultrasmedbio.2013.10.015 24462154

[48] Wortsman X Jemec GBE Dermatologic Ultrasound with Clinical and Histologic Correlations. New York: Springer (2013).

[49] Chami L Lassau N Chebil M Robert C. Imaging of melanoma: usefulness of ultrasonography before and after contrast injection for diagnosis and early evaluation of treatment. Clin Cosmet Investig Dermatol (2011) 4:1–6. 10.2147/CCID.S13499 21673868 PMC3108283

[50] Rubaltelli L Beltrame V Scagliori E Bezzon E Frigo AC Rastrelli M Potential use of contrast-enhanced ultrasound (CEUS) in the detection of metastatic superficial lymph nodes in melanoma patients. Ultraschall Med (2014) 35:67–71. 10.1055/s-0033-1335857 23860858

[51] Crişan M Crişan D Sannino G Lupsor M Badea R Amzica F. Ultrasonographic staging of cutaneous malignant tumors: an ultrasonographic depth index. Arch Dermatol Res (2013) 305:305–13. 10.1007/s00403-013-1321-1 23400334

[52] Martorell A Segura Palacios JM Ultrasound examination of hidradenitis suppurativa. Actas Dermosifillogr (2015) 106(Suppl 1):49–59. 10.1016/S0001-7310(16)30007-2 26895939

[53] Martorell A Wortsman X Alfageme F Roustan G Arias-Santiago S Catalano O Ultrasound evaluation as a complementary test in hidradenitis suppurativa: proposal of a standardized report. Dermatol Surg (2017) 43:1065–73. 10.1097/DSS.0000000000001147 28538033

[54] Newman TM Sheth MM Levy A Babagbemi K Hidradenitis suppurativa: mammographic and sonographic manifestations in two cases. Radiol Case Rep (2013) 8:737 10.2484/rcr.v8i3.737 27330633 PMC4900121

[55] Zarchi K Jemec GB The role of ultrasound in severity assessment in hidradenitis suppurativa. Dermatol Surg (2014) 40:592 10.1111/dsu.12437 24446665

[56] Jemec GB Gniadecka M. Ultrasound examination of hair follicles in hidradenitis suppurativa. Arch Dermatol (1997) 133:967–70. 10.1001/archderm.133.8.967 9267241

[57] Wortsman X Jemec GB. Real-time compound imaging ultrasound of hidradenitis suppurativa. Dermatol Surg (2007) 33:1340–2. 10.1097/00042728-200711000-00007 17958586

[58] Wortsman X Moreno C Soto R Arellano J Pezo C Wortsman J. Ultrasound in-depth characterization and staging of hidradenitis suppurativa. Dermatol Surg (2013) 39:1835–42. 10.1111/dsu.12329 24118433

[59] Kelekis NL Efstathopulos E Balanika A Spyridopoulos TN Pelekanou A Kanni T Ultrasound aids in diagnosis and severity assessment of hidradenitis suppurativa. Br J Dermatol (2010) 162:1400–2. 10.1111/j.1365-2133.2010.09710.x 20184586

[60] Zarchi K Yazdanyar N Yazdanyar S Wortsman X Jemec GB. Pain and inflammation in hidradenitis suppurativa correspond to morphological changes identified by high-frequency ultrasound. J Eur Acad Dermatol Venereol (2015) 29:527–32. 10.1111/jdv.12610 25124135

[61] Wortsman X Wortsman J Ultrasound detection of retained hair tracts in hidradenitis suppurativa. Dermatol Surg (2015) 41:867–9. 10.1097/DSS.0000000000000388 26050213

[62] Wortsman X Rodriguez C Lobos C Eguiguren G Molina MT. Ultrasound diagnosis and staging in pediatric hidradenitis suppurativa. Pediatr Dermatol (2016) 33:e260–4. 10.1111/pde.12895 27292973

[63] Wortsman X. Imaging of hidradenitis suppurativa. Dermatol Clin (2016) 34:59–68. 10.1016/j.det.2015.08.003 26617359

[64] Kolodziejczak M Sudoł-Szopińska I Wilczyńska A Bierca J. Utility of transperineal and anal ultrasonography in the diagnostics of hidradenitis suppurativa and its differentiation from a rectal fistula. Postepy Hig Med Dosw (Online) (2012) 66:838–42. 10.5604/17322693.1019537 23175339

[65] Gurjarpadhye AA Parekh MB Dubnika A Rajadas J Inayathullah M. Infrared Imaging tools for diagnostic applications in dermatology. SM J Clin Med Imaging (2015) 1:1–5. 26691203 PMC4683617

[66] Borsari S Pampena R Lallas A Kyrgidis A Moscarella E Benati E Clinical indications for use of reflectance confocal microscopy for skin cancer diagnosis. JAMA Dermatol (2016) 152:1093–8. 10.1001/jamadermatol.2016.1188 27580185

[67] Mogensen M Joergensen TM Nürnberg BM Morsy HA Thomsen JB Thrane L Assessment of optical coherence tomography imaging in the diagnosis of non-melanoma skin cancer and benign lesions versus normal skin: observer-blinded evaluation by dermatologists and pathologists. Dermatol Surg (2009) 35:965–72. 10.1111/j.1524-4725.2009.01164.x 19397661

[68] Stanganelli I Longo C Mazzoni L Magi S Medri M Lanzanova G Integration of reflectance confocal microscopy in sequential dermoscopy follow-up improves melanoma detection accuracy. Br J Dermatol (2015) 172:365–71. 10.1111/bjd.13373 25154446

[69] Stevenson AD Mickan S Mallett S Ayya M. Systematic review of diagnostic accuracy of reflectance confocal microscopy for melanoma diagnosis in patients with clinically equivocal skin lesions. Dermatol Pract Concept (2013) 3:19–27. 10.5826/dpc.0304a05 24282659 PMC3839827

[70] Grazziotin TC Alarcon I Bonamigo RR Carrera C Potrony M Aguilera P Association between confocal morphologic classification and clinical phenotypes of multiple primary and familial melanomas. JAMA Dermatol (2016) 152:1099–105. 10.1001/jamadermatol.2016.1189 27579522

[71] Maher NG Solinas A Scolyer RA Guitera P In vivo reflectance confocal microscopy for evaluating melanoma of the lip and its differential diagnoses. Oral Surg Oral Med Oral Pathol Oral Radiol (2017) 123:84–94. 10.1016/j.oooo.2016.08.011 27720652

[72] Xiang W Peng J Song X Xu A Bi Z. Analysis of debrided and non-debrided invasive squamous cell carcinoma skin lesions by in vivo reflectance confocal microscopy before and after therapy. Lasers Med Sci (2017) 32:211–9. 10.1007/s10103-016-2104-7 27837338

[73] Alarcon I Pasquali P Malvehy J Puig S Tumor regrowth and development of keratinocytic neoplasms in patients under smoothened inhibition: in vivo assessment with reflectance confocal microscopy. Skin Res Technol (2016) 23:283–8. 10.1111/srt.12332 27785832

[74] Chuah SY Tee SI Tan WP Lee SSJ Ng SK Chua SH Reflectance confocal microscopy is a useful non-invasive tool in the in vivo diagnosis of pigmented basal cell carcinomas in Asians. Australas J Dermatol (2015) 58:130–4. 10.1111/ajd.12401 26390992

[75] Kadouch DJ Schram ME Leeflang MM Limpens J Spuls PI de Rie MA. In vivo confocal microscopy of basal cell carcinoma: a systematic review of diagnostic accuracy. J Eur Acad Dermatol Venereol (2015) 29:1890–7. 10.1111/jdv.13224 26290493

[76] Nelson SA Scope A Rishpon A Rabinovitz HS Oliviero MC Laman SD Accuracy and confidence in the clinical diagnosis of basal cell using dermoscopy and reflex confocal microscopy. Int J Dermatol (2016) 55:1351–6. 10.1111/ijd.13361 27419915

[77] Astner S González S Cuevas J Röwert-Huber J Sterry W Stockfleth E Preliminary evaluation of benign vascular lesions using in vivo reflectance confocal microscopy. Dermatol Surg (2010) 36:1099–100. 10.1111/j.1524-4725.2010.01590.x 20653723

[78] Friedman D Friedman PC Gill M. Reflectance confocal microscopy: an effective diagnostic tool for dermatophytic infections. Cutis (2015) 95:93–7. 25750962

[79] Welzel J Lankenau E Birgurber R Engelhardt R. Optical coherence tomography of the human skin. J Am Acad Dermatol (1997) 37:958–63. 10.1016/S0190-9622(97)70072-0 9418764

[80] Varkentin A Mazurenka M Blumenröther E Meinhardt-Wollweber M Rahlves M Broekaert SM Comparative study of presurgical skin infiltration depth measurements of melanocytic lesions with OCT and high frequency ultrasound. J Biophotonics (2016) 10:854–61. 10.1002/jbio.201600139 28009131

[81] Cheng HM Lo S Scolyer R Meekings A Carlos G Guitera P. Accuracy of optical coherence tomography for the diagnosis of superficial basal cell carcinoma: a prospective, consecutive, cohort study of 168 cases. Br J Dermatol (2016) 175:1290–300. 10.1111/bjd.14714 27146027

[82] Ulrich M von Braunmuehl T Kurzen T Dirschka T Kellner C Sattler E The sensitivity and specificity of optical coherence tomography for the assisted diagnosis of nonpigmented basal cell carcinoma: an observational study. Br J Dermatol (2015) 173:428–35. 10.1111/bjd.13853 25904111

[83] Gambichler T Schmid-Wendtner MH Plura I Kampilafkos P Stücker M Berking C A multicentre pilot study investigating high-definition optical coherence tomography in the differentiation of cutaneous melanoma and melanocytic nevi. J Eur Acad Dermatol Venereol (2015) 29:537–41. 10.1111/jdv.12621 25073788

[84] Schuh S Holmes J Ulrich M Themstrup L Jemec GBE De Carvalho N Imaging blood vessel morphology in skin: dynamic optical coherence tomography as a novel potential diagnostic tool in dermatology. Dermatol Ther (2017) 7:187–202. 10.1007/s13555-017-0175-4 28258554 PMC5453917

[85] Olsen J Themstrup L Jemec GB. Optical coherence tomography in dermatology. G Ital Dermatol Venereol (2015) 150:603–15. 10.1117/1.JBO.18.6.061224 26129683

[86] Matts PJ Cotton SD Spectrophotometric intracutaneous analysis (SIAscopy). Chapter 25. 3rd ed In: Barel AO Paye M Maibach HI, editors. Handbook of Cosmetic Science and Technolgoy. New York, NY: Informa Healthcare USA, Inc. (2009). p. 275–81.

[87] Malvehy J Hauschild A Curiel-Lewandrowski C Mohr P Hofmann-Wellenhof R Motley R Clinical performance of the Nevisense system in cutaneous melanoma detection: an international, multicenter, prospective and blinded clinical trial on efficacy and safety. Br J Dermatol (2014) 171:1099–107. 10.1111/bjd.13121 24841846 PMC4257502

[88] Glud M Gniadecki R Drzewiecki KT. Spectrophotometric intracutaneous analysis versus dermoscopy for the diagnosis of pigmented skin lesions: prospective, double-blind study in a secondary reference centre. Melanoma Res (2009) 19:176–9. 10.1097/CMR.0b013e328322fe5f 19319002

[89] Vestergaard ME Macaskill PHPM Holt PE Menzies SW. Dermoscopy compared with naked eye examination for the diagnosis of primary melanoma: a meta-analysis of studies performed in a clinical setting. Br J Dermatol (2008) 159:669–76. 10.1111/j.1365-2133.2008.08713.x 18616769

[90] VivoSight. VivoSight. Michelson Diagnostics. (2015). Available from: https://us.vivosight.com (Accessed: February 12, 2018).

[91] Blue Cross Blue Shield Rhode Island (BCBSRI). Medical coverage policy – reflectance confocal microscopy for evaluating skin lesions for suspected malignancy. BCBSRI (2016). Available from: https://www.bcbsri.com/sites/default/files/polices/Reflectance%20Confocal%20Microscopy_Final%20V1.1_0.pdf (Accessed: February 19, 2018).

[92] Health Canada. Medical devices active licences search. Gov Can (2018). Available from: https://health-products.canada.ca/mdall-limh/dispatch-repartition.do?type=active (Accessed: February 20, 2018).

[93] U.S. Food and Drug Administration. Establishment registration & device listing. Health & Human Services. (2018). Available from: https://www.accessdata.fda.gov/scripts/cdrh/cfdocs/cfRL/TextSearch.cfm (Accessed: February 20, 2018).

[94] Esteva A Kuprel B Novoa RA Ko J Swetter SM Blau HM Dermatologist-level classification of skin cancer with deep neural networks. Nature (2017) 542:115–8. 10.1038/nature21056 28117445 PMC8382232

[95] Young SR Bolton PA Downie J. Use of high-frequency ultrasound in the assessment of injectable dermal fillers. Skin Res Technol (2008) 14:320–3. 10.1111/j.1600-0846.2008.00297.x 19159378

[96] Hofmann M Saigo R Aschoff R Luger TA Meurer M Bräutigam M Validation of Dermaphot^®^ for the assessment of steroid-induced skin atrophy. Arch Dermatol Res (2013) 305:215–21. 10.1007/s00403-012-1297-2 23242470

[97] Moore JV Allan E Therapeutics – pulsed ultrasound measurements of depth and regression of basal cell carcinomas after photodynamic therapy: relationship to probability of 1-year local control. Br J Dermatol (2003) 149:1035–40. 10.1111/j.1365-2133.2003.05558.x 14632811

[98] Ballester-Sánchez R Pons-Llanas O Llavador-Ros M Botella-Estrada R Ballester-Cuñat A Tormo-Micó A Depth determination of skin cancers treated with superficial brachytherapy: ultrasound vs. histopathology. J Contemp Brachytherapy (2015) 6:356–61. 10.5114/jcb.2014.47860 25834579 PMC4300362

[99] Kang T Abignano G Lettieri G Wakefield RJ Emery P Del Galdo F. Skin imaging in systemic sclerosis. Eur J Rheumatol (2014) 1:111–6. 10.5152/eurjrheumatol.2014.036 27708890 PMC5042219

[100] Porriño-Bustamante ML Alfageme F Suárez L de Domingo MA Hospital M Roustán G. High-frequency color Doppler sonography of bullous pemphigoid: correlation with histologic findings. J Ultrasound Med (2016) 35:1821–5. 10.7863/ultra.15.09079 27371374

